# Herlyn Werner Wunderlich Syndrome with Hematocolpos: An Unusual Case Report of Full Diagnostic Approach and Treatment 

**DOI:** 10.22074/ijfs.2016.4779

**Published:** 2016-04-05

**Authors:** Rohit Bhoil, Ajay Ahluwalia, Narvir Chauhan

**Affiliations:** Department of Radiodiagnosis, Dr. Rajendra Prasad Government Medical College, Kangra, HP, India

**Keywords:** Amenorrhea, Dysmenorrhea, Hematocolpos, Vagina, Infertility

## Abstract

Herlyn-Werner-Wunderlich (HWW) syndrome is an uncommon combined müllerian duct anomalies (MDAs) and mesonephric duct malformation of female urogenital tract characterized by uterus didelphys and obstructed hemi-vagina and ipsilateral renal agenesis (OHVIRA) syndrome. We present a rare and unusual case of this syndrome in a 19 year-old female who suffered from hypomenorrhoea and abdominal pain. She had an obstructed hemi-vagina on right side which led to marked distention of ipsilateral cervix, while proximal hemi-vagina compressed the contralateral side causing its partial obstruction resulting in hypomenorrhoea. Understanding the imaging findings of this rare condition is important for early diagnosis in order to prevent complications which may lead to infertility.

## Introduction

Herlyn Werner Wunderlich (HWW) syndrome, also known as obstructed hemi-vagina and ipsilateral renal agenesis (OHVIRA) syndrome, is an uncommon combined müllerian duct anomalies (MDAs) (Table 1) and mesonephric duct malformation of female urogenital tract.

**Table 1:** MDAs classification

Description	Class
I	Segmental müllerian agenesis or hypoplasia
II	Unicornuate uterus
III	Uterus didelphys
IV	Bicornuate uterus
V	Septate uterus
VI	Arcuate uterus
VII	Uterus with internal luminal changes (T shaped uterus - diethylstilboestrol exposure related)

MDAs; Müllerian duct anomalies.

**Fig.1:**
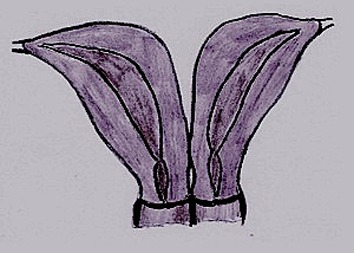
Class III MDAs-uterine didelphys. MDAs; Müllerian duct anomalies.

The exact incidence of this syndrome is unknown (1); however, the incidence of uterus didelphys (Fig .1) as a part of this syndrome is about 1/2000 to 1/28000 that is accompanied by unilateral renal agenesis with the incidence of approximately 1/1100, while 25 to 50% of affected women have showed to have genital abnormalities (2-5).

Although HWW syndrome includes variability of the anatomic structures like uterine, cervical, vaginal and/or renal anomalies, it is characterized by the presence of uterus duplicity and OHVIRA syndrome.

## Case report

A 19 year-old unmarried female presented to Dr. Rajendra Prasad, Government Medical College, Kangra, HP, India, in June 2013. She complained of abdominal pain gradually increasing in intensity and scanty periods since the last 6 months. Patient reached menarche at 16 years with normal menstrual cycles until 6 months ago. She also complained of periodic pain in lower abdomen accompanying her menstrual cycles beginning from around the time of her menarche. Initially for the first three-four months, she was being symptomatically managed for dysmenorrhea, but ultrasound scans done in a referral center revealed multiple cystic lesions in bilateral adnexa with low level internal echoes suggestive of endometriosis. Thereafter she was being managed medically for endometriosis (in the scans, her uterus was reported as normal). Her urine pr egnancy test was negative.

**Fig.2:**
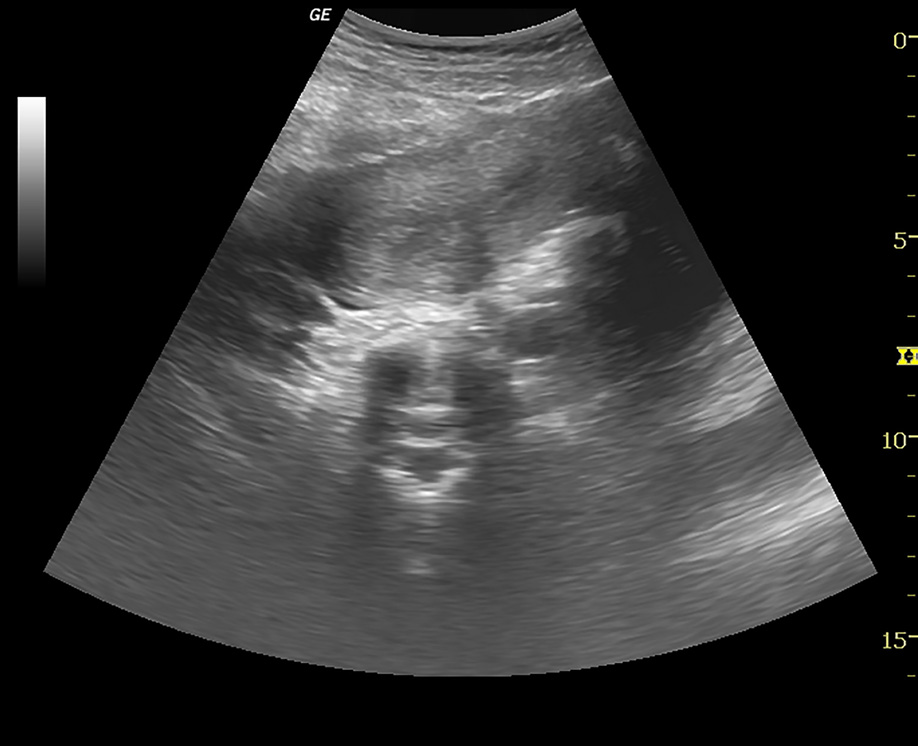
Transverse ultrasound image showing two uterine cavities with echogenic endometrium.

**Fig.3:**
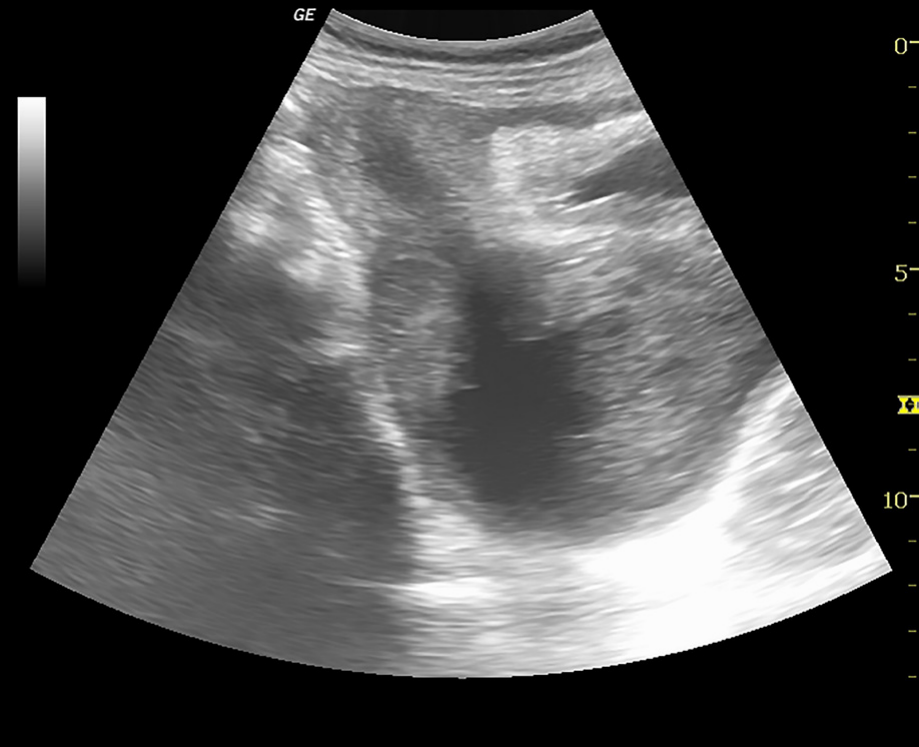
Longitudinal ultrasound image depicting a cystic lesion posterior to urinary bladder with low level echoes and communication with endometrial cavity through the cervix.

**Fig.4:**
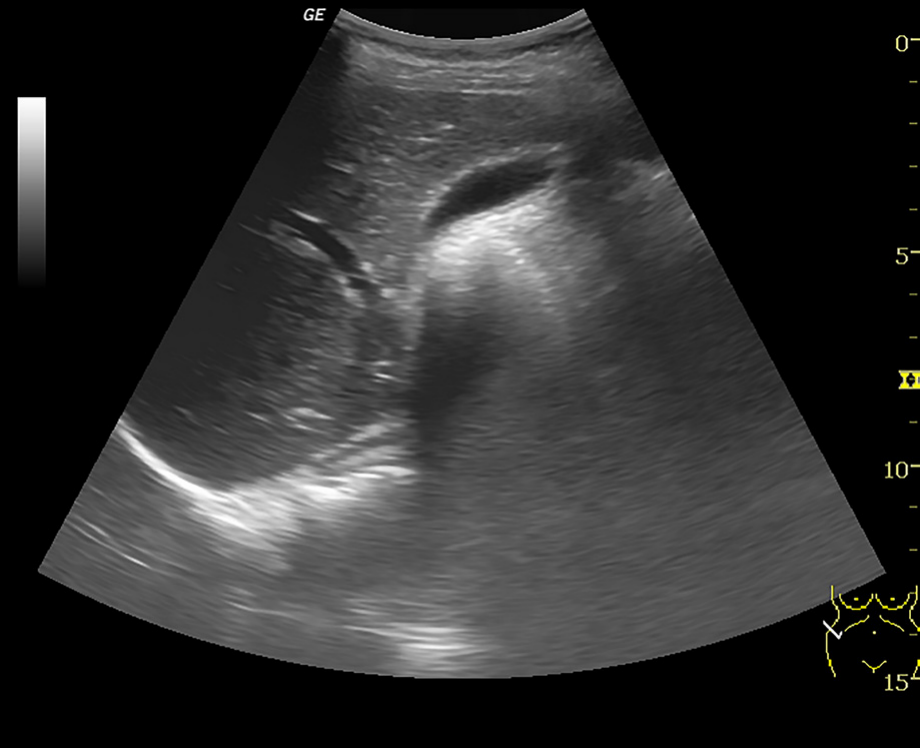
Transverse ultrasound of right hepatorenal space showing absent kidney in the right renal fossa.

An ultrasound scan done for pelvic organs at our institute revealed uterus didelphys (Fig .2) and a cystic fluid collection with low level internal echoes arising from the pelvis consistent with associated haematocolpos (Fig .3). Cystic lesion was noted in the right adnexa consistent with endometrioma. Right kidney was not visualized (Fig .4). 

**Fig.5:**
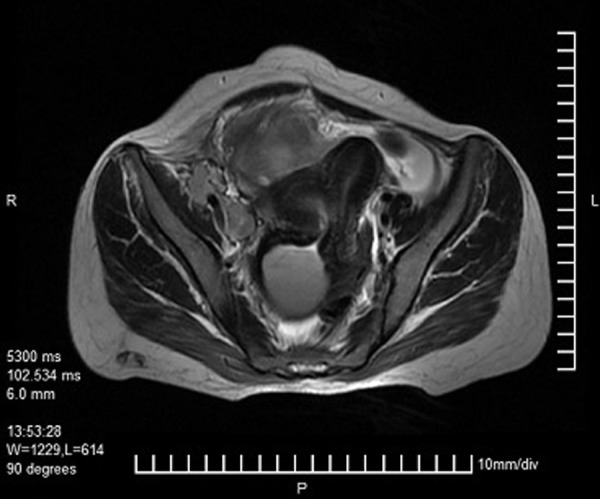
Axial T2W MRI image showing two uterine cavities with distended right cervix and hemi-vagina. MRI; Magnetic resonance imaging.

**Fig.6:**
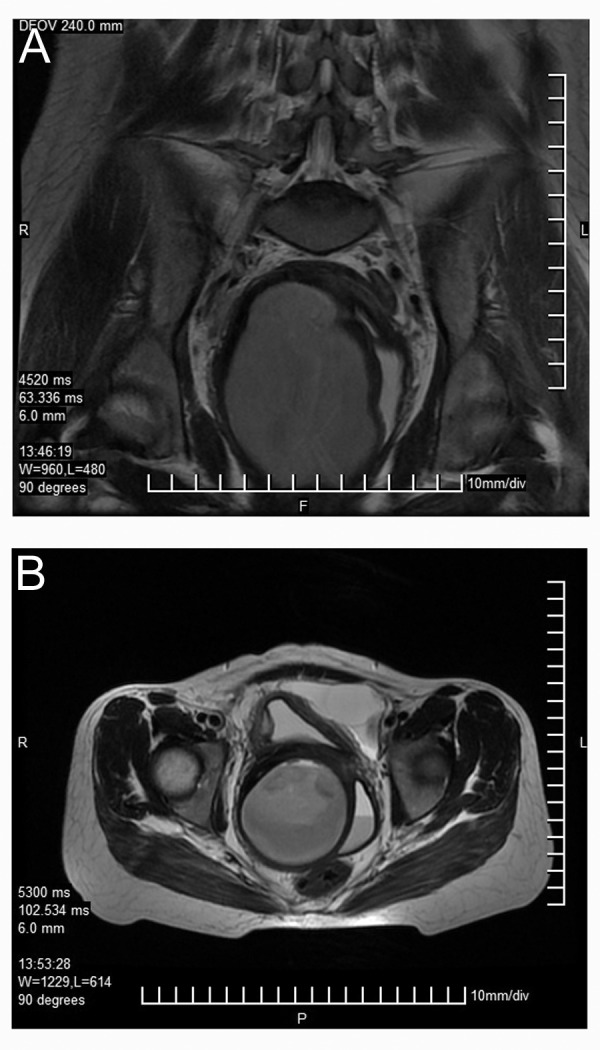
A. Coronal and B. Axia T2W MRI images showing distended right cervix and hemi-vagina compressing the normal left hemi-vagina which shows differential signal intensity resulting in layering.

Subsequently magnetic resonance imaging (MRI) was performed to better characterize the pelvic anatomy and better identify the anatomic location of this pelvic fluid collection. MRI revealed uterine didelphys with two separate cervices (Fig .5). The right cervix and proximal hemi-vagina were distended that led to the comparison of the left cervix and hemi-vagina (Fig .6A). The left endometrial cavity appeared normal; however, the left cervix/ hemi-vagina was constricted in its lower part due to pressure from the distended cervix and hemi-vagina on the right side resulting in partial obstruction of menstrual blood outflow, as seen in the patient (Fig .6B). The high T2 MRI signal characteristics in Transverse ultrasound of right hepatorenal space showing absent kidney in the right renal fossa.

**Fig.7:**
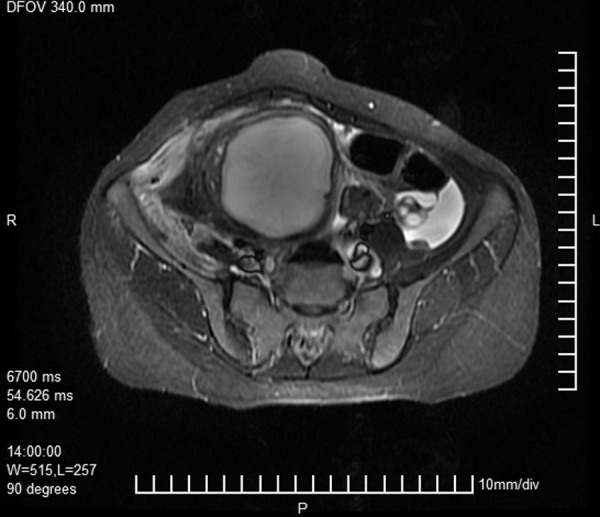
Axial T2W MRI image showing large hyperintense right adnexal cyst (endometriotic cyst). MRI; Magnetic resonance imaging.

A right adnexal cystic lesion with blood products was seen suggestive of endometriotic cyst (Fig .7).

Subsequent gynecological examination revealed an obstructed right hemi-vagina and a fluid wave palpable through inferior septum. This hematocolpos was surgically drained and about 400 ml of old blood was evacuated. The patient recovered uneventfully and no further surgery was done.

A written consent was taken from the patient for publication of this report.

## Discussion

Most common type of MDAs is the lateral fusion defects which range from symmetric/asymmetric to obstructed/unobstructed fusion anomalies. A useful classification based on the degree of failure of normal development was proposed by Buttram and Gibbons (6).

Development of urinary system and müllerian duct system are closely related with which accounts for the frequent association of anomalies involving both the systems (2, 3).

Uterine didelphys results from complete failure of fusion of the müllerian ducts and their normal differentiation to form a cervix and uterus during the 8th week of gestation (7). Uterine didelphys (Class III MDA) occurs in case of complete failure of fusion as also seen in our case. 

The Wolffian duct gives rise to the ipsilateral ureteric bud and thus is responsible for the formation of the kidney. Accordingly, in the absence of the Wolffian duct on one side, the kidney and ureter (of the same side) will fail to fuse (3,4). On the side on which the Wolffian duct is missing, the müllerian duct is displaced laterally and fails to adequately fuse with the urogenital sinus, leading to the formation of a blind sac, imperforate or obstructed hemivagina (3), right side in the present case. The distal part of vagina which arises from the urogenital sinus is not affected and develops normally. 

Patients with OHVIRA syndrome are usually asymptomatic until puberty, when they present with acute lower abdominal pain. Diagnosis is usually made soon after menarche (most patients are diagnosed from 2 months to 2 year after menarche) and the presenting symptoms are pelvic pain, dysmenorrhea, foul-smelling discharge and pelvic mass (7,8). If not treated, complications leading to infertility, endometriosis, pelvic adhesions, and pyosalpinx or pyocolpos may present in the late phase with a high miscarriage rate (7). 

The choice of investigation for the diagnosis and operative planning of OHVIRA syndrome are ultrasound and MRI, both of which have an added advantage of being non-invasive (1,5). 

The role of computed tomography (CT) is limited due to radiation exposure and limited soft-tissue resolution. Ultrasound may reveal uterine didelphus and pelvic fluid collection with low level internal echoes, contiguous with the endocervix (haemato/pyocolpos). Due to retrograde menstruation, features of endometriosis in form of well defined, unilocular or multilocular, predominantly cystic masses containing diffuse, homogeneous, low level internal echoes (endometrioma/chocolate cyst) may also be seen (9). 

MRI plays an important role in characterizing the didelphic uterus, obstructed hemivagina, and ipsilateral renal agenesis (1,10). MRI findings of OHVIRA syndrome are characterized by iso/high T1W signal and high T2W signal that indicate pelvic fluid collection is contiguous with the endocervix along with didelphic uterus and an absent kidney on the affected side (1,2). 

MRI is far better than ultrasound for characterizing anatomical relationships due to its multiplanar capabilities and larger field of view (2). However, the gold standard for diagnosis is laproscopy through which has the added benefit of performing therapeutic drainage of hematometra/hematocolpos, vaginal septotomy and marsupialisation (10). Treatment usually involves surgery in the form of excision of the vaginal septum which helps in relieving obstruction (11). Surgical intervention also decreases the chances of pelvic endometriosis due to retrograde menstrual seeding. About 87% of patients go on to have a successful pregnancy; however, 23% of patients carry the risk of subsequent abortion (12). 

The rarity of OHVIRA syndrome complicates its diagnosis, and hence clinicians and radiologists should consider MDAs among the differential diagnosis in young female patients presenting with abdominal symptoms, especially when associated with renal anomaly/agenesis. Understanding the imaging findings is critical for early diagnosis in an attempt to prevent complications such as endometriosis or adhesions from chronic infections with subsequent infertility. 
